# The role of neural reward sensitivity in the longitudinal relations between parents’ familism values and Latinx American youth’s prosocial behaviors

**DOI:** 10.1016/j.dcn.2024.101343

**Published:** 2024-01-15

**Authors:** Beiming Yang, Zexi Zhou, Varun Devakonda, Yang Qu

**Affiliations:** a School of Education and Social Policy, Northwestern University, USA; b Department of Human Development and Family Sciences, University of Texas at Austin, USA

**Keywords:** Adolescence, Familism, Latinx, Prosocial behavior, Reward sensitivity

## Abstract

Past research suggests that parents’ familism values play a positive role in Latinx American youth’s prosocial tendencies. However, little is known about how individual differences in youth’s neural development may contribute to this developmental process. Therefore, using two-wave longitudinal data of 1916 early adolescents (mean age = 9.90 years; 50% girls) and their parents (mean age = 38.43 years; 90% mothers) from the Adolescent Brain Cognitive Development study, this pre-registered study took a biopsychosocial approach to examine the moderating role of youth’s neural reward sensitivity in the link between parents’ familism values and youth’s prosocial behaviors. Results showed that parents’ familism values were associated with increased prosocial behaviors among youth two years later, controlling for baseline prosocial behaviors and demographic covariates. Notably, parents’ familism values played a larger role in promoting youth’s prosocial behaviors among youth who showed lower ventral striatum activation during reward anticipation. Moreover, such association between parents’ familism values and youth’s later prosocial behaviors was stronger among youth who showed lower levels of prosocial behaviors initially. Taken together, the findings highlight individual differences in neurobiological development and baseline prosocial behaviors as markers of sensitivity to cultural environments with regard to Latinx American youth’s prosocial development.

## Highlights

• Parents’ familism values predict youth’s increased prosocial behaviors over time.
• Youth show neural and behavioral differential susceptibility to familism values.
• Youth with lower neural reward sensitivity are more sensitive to familism.
• Youth with lower prosocial behaviors initially are more likely to be influenced.

In the United States, Latinx Americans are the largest and fastest growing ethnic minority, making up nearly twenty percent of the population. In recent years, increasing attention has been paid to cultural values, such as familism, as a protective factor for Latinx American youth’s positive development (Cahill et al., 2021). Given that familism places an emphasis on helping other family members on a regular basis (Sabogal et al., 1987), one of the positive developmental outcomes that is culturally valued among Latinx American youth is prosocial behaviors – actions intended to benefit others (Carlo and de Guzman, 2009, Knight and Carlo, 2012). Although prior research has examined the positive role of parents’ familism values in youth’s prosocial development (Calderón-Tena et al., 2011, Knight et al., 2016), less is known about what factors may moderate this link. Given the individual variability in youth’s neural sensitivity to social environments (Guyer, 2020, Schriber and Guyer, 2016), the role of parents’ familism values in youth’s prosocial behaviors may vary depending on individual differences in youth’s brain development. In particular, neural reward sensitivity may moderate the link between parents’ familism values and youth’s prosocial behavior. Moreover, prior research suggests that parental socialization beliefs tend to have a larger impact on youth’s prosocial development over time among youth who initially show a lower level of prosocial behaviors (Zhou et al., 2022). Therefore, using longitudinal data from the Latinx American sample of the Adolescent Brain Cognitive Development (ABCD) study, the current study aimed to investigate the longitudinal association between parents’ familism values and youth’s prosocial behaviors over two years during early adolescence, with attention to the moderating roles of individual differences in youth’s neural (i.e., neural reward sensitivity) and behavioral (i.e., baseline prosocial behaviors) development.

Familism is a core value of Latinx culture, which is characterized by providing support (i.e., family support), fulfilling obligations (i.e., family obligation), and showing solidarity to family members (i.e., family as referent) (Knight et al., 2010, Sabogal et al., 1987). Familism’s protective role in child development is most evident during late childhood and adolescence (Stein et al., 2014). Cultural socialization theories indicate that parents tend to socialize their children to acquire specific qualities and behaviors valued by the cultural contexts (Knight et al., 1993, Umaña‐Taylor et al., 2009). Therefore, Latinx American parents who endorse greater familism values may raise youth who are more likely to develop prosocial behaviors given that such behaviors are adaptive in the cultural environment of valuing others (Knight and Carlo, 2012). Moreover, parents who endorse greater familism values are more likely to ask their youth to fulfill family responsibilities such as taking care of younger siblings or relatives (Calderón-Tena et al., 2011). Such practices may promote youth’s awareness, consideration, and responsiveness to other family members’ needs, which positively contribute to their prosocial development (Knight et al., 2015, Knight et al., 2018). Indeed, prior concurrent and longitudinal studies on Mexican American families have documented positive associations between parents’ familism values and youth’s prosocial tendencies (Calderón-Tena et al., 2011, Knight et al., 2016). However, less is known about how such associations may vary depending on youth’s individual differences. Given the enormous variability among Latinx American youth, it is important to further explore what factors may moderate the link between parents’ familism values and youth’s prosocial behaviors. Advances in this line will help identify youth who may benefit more from culturally informed interventions, which will ultimately contribute to Latinx American youth’s positive development.

Theories on adolescent brain development suggest that individual differences in neurobiological development can moderate the impact of social contexts (e.g., cultural, parental, and peer factors) on youth’s developmental outcomes ranging from psychological, behavioral, to academic adjustment (Guyer, 2020, Schriber and Guyer, 2016). In this vein, Latinx American youth’s brain development may serve as an important marker of susceptibility to parents’ familism values. According to the differential susceptibility model (Belsky et al., 2007, Ellis and Boyce, 2011), Latinx youth characterized by heightened susceptibility to familism may be more likely to be influenced by familism in both a positive and negative way (i.e., high familism as enhancement and low familism as vulnerability), whereas youth who have lower susceptibility to familism may be less likely to be influenced by such cultural values. Youth’s neural reward sensitivity is a possible marker of neurobiological susceptibility to social contexts. Specifically, a neural region that is central to reward processing is the ventral striatum. During early adolescence, individual differences in the ventral striatum play a vital role in multiple aspects of development such as mental health, cognitive persistence, and risk-taking behaviors (for a review, see Telzer, 2016). In both humans (Somerville et al., 2010) and rodents (Spear, 2011), early adolescents exhibit enhanced novelty and incentive-seeking behaviors, which may be contributed by heightened ventral striatum activity during reward processing (Crone et al., 2016, Telzer, 2016, Van Duijvenvoorde et al., 2016).

During adolescence, heightened sensitivity to monetary reward may reflect youth’s worse relationships with parents, which is key for the transmission of cultural values. Given that early adolescence is a period when children start to individuate from parents (Levpušček, 2006, McLean et al., 2010), there are significant individual differences in parent-child relationships during this period. Past research suggests that youth’s worse relationships with parents (e.g., decreased disclosure and increased conflict) are associated with their increased ventral striatum response to reward (Qu et al., 2015). Similarly, parents’ negative attitudes toward youth also contribute to youth’s increased ventral striatum to reward over time (Casement et al., 2014). Youth with worse relationships with parents may receive less positive feedback from their parents, and thus they may seek rewards outside the family such as monetary rewards to compensate for the lack of social rewards at home (Qu et al., 2015). Past research suggests that worse parent-child relationships hinder parents’ socialization of cultural values (Tsai et al., 2015). Therefore, when Latinx American youth are highly attuned to monetary reward, they may be less receptive to their parents’ socialization of familism values. For them, it is more difficult for parents’ familism values to play a role in their prosocial development. In contrast, Latinx American youth’s low sensitivity to monetary reward may reflect better relationships with parents (Qu et al., 2015), which helps them internalize parents’ cultural values. Therefore, their prosocial behaviors are more likely to be influenced by parents’ familism values.

Youth’s baseline prosocial behaviors also may moderate the links between parents’ familism values and youth’s prosocial behaviors. Past research suggests that youth in a less adaptive status may have a heightened need for parental resources (Pomerantz et al., 2007). In the case of prosocial development, youth in a less adaptive status (i.e., low prosociality) may lack the ability and motivation to engage in prosocial behaviors. For them, their prosocial behaviors may highly depend on the social environment they live in. Therefore, youth who initially exhibit a lower level of prosocial behaviors may be more sensitive to parents’ socialization beliefs and practices, which can provide developmental resources for prosocial development that are especially needed by these youth. In contrast, given that youth with greater prosocial behaviors at baseline have already established a high level of prosociality, parents are unlikely to further promote their prosocial behaviors. Indeed, a recent study suggested that parents’ collectivism socialization goals (i.e., expectations for children to have harmonious relationships and develop interdependency with others) predicted increased Chinese adolescents’ prosocial behaviors over time only among those who reported a lower baseline level of prosocial behaviors, but not among those who reported a higher baseline level of prosocial behaviors (Zhou et al., 2022). Therefore, Latinx American parents’ familism values also may play a larger role in their youth’s prosocial development when youth initially show a lower level of prosocial behaviors.

## 1 Current study

Using longitudinal data from the ABCD study, the current research aimed to examine the longitudinal association between parents’ familism values and youth’s prosocial behaviors, with attention to the moderating role of youth’s neural reward sensitivity and baseline prosocial behaviors. Youth’s neural reward sensitivity was measured by ventral striatum activation during reward processing in the Monetary Incentive Delay task (MID task, Knutson et al., 2001; Knutson and Heinz, 2015; Yau et al., 2012). Given that both reward anticipation and reward receipt exhibit age-specific uniqueness during adolescence (Van Leijenhorst et al., 2010) and both are relevant for adolescent development (Forbes et al., 2010) , ventral striatum activation during both phases was included in the current study. The hypotheses and analyses were pre-registered (https://aspredicted.org/YCL_PBR). Guided by prior research (Calderón-Tena et al., 2011, Zhou et al., 2022), we had the following hypotheses. First, we hypothesized that parents’ familism values (i.e., a latent variable indicated by family support, family obligation, and family as referent; Knight et al., 2016; Streit et al., 2021) may predict increased prosocial behaviors two years later among Latinx American youth, after controlling for youth’s prosocial behaviors at baseline and demographic covariates. Second, we hypothesized that the longitudinal association between parents’ familism values and youth’s prosocial behaviors may be moderated by youth’s neural reward sensitivity, such that parents’ familism values may play a larger role in Latinx American youth’s prosocial behaviors over time among youth who show lower neural reward sensitivity. Finally, we hypothesized that the longitudinal association between parents’ familism values and youth’s prosocial behaviors may be moderated by youth’s initial prosocial behaviors, such that parents’ familism values may only predict youth’s increased prosocial behaviors among youth who initially show a lower level of prosocial behaviors.

## 2 Methods

### 2.1 Participants

Data were obtained from baseline (T1) and two-year follow-up (T2) of the Adolescent Brain Cognitive Development (ABCD) study (data release 4.0). All the data included in the current study are available on the NIMH Data Archive (https://nda.nih.gov/abcd) upon data access request. Participants of the ABCD study were recruited at 21 sites in the United States using probability sampling (Garavan et al., 2018). Previous work documents a variety of measures that were used for this study, including task-based fMRI and behavioral outcomes (Casey et al., 2018). Among the full Latinx American sample of 2411 youth at T1, a total of 1916 Latinx American youth (mean age = 9.90 years, SD =.63 years; 50% girls) and their primary caregivers (90% mothers) were included in the analyses. 94% of the youth and 53% of the parents were born in the United States. The current research included participants based on the inclusion criteria provided by the ABCD team (i.e., participants with variable “imgincl_mid_include” = 1), which are the recommended quality control criteria of the MID task in ABCD data release note 4.0 (e.g., passing the MID task behavior cutoff, FreeSurfer quality control, and fMRI manual post-processing quality control; for detailed criteria, see ABCD Human Subjects Study, 2021). Among the full Latinx American sample of 2411 youth at baseline, 495 youth were excluded for neuroimaging quality control purposes. Independent samples t-test showed that youth who were excluded for quality control had younger age (*p* < .001), were more likely to be boys (*p* < .001), and had lower parental educational attainment (*p* = .002). No differences were found in other demographic characteristics (i.e., parents’ gender, nativity, and household financial adversity) or key variables of this study (i.e., parents’ familism values and youth’s prosocial behaviors).

### 2.2 The monetary incentive delay (MID) task

At T1, youth’s ventral striatum activity during reward processing was acquired from tabulated and region of interest-based results of the Monetary Incentive Delay (MID) task of the ABCD study. In the MID task (Knutson et al., 2001, Yau et al., 2012), the participant attempted to win money by rapidly pressing a button. Each trial included three relevant epochs including an anticipation phase, where the participant was informed if the current trial was a ‘win’ or ‘lose’ trial, a motor period, where the participant rapidly pressed a button in response to a prompt, and an outcome phase, where the participant was informed how they performed. There were three types of trials in the MID task. On ‘win’ trials, the participant can win money or fail to win money depending on their performance. On ‘lose’ trials, the participant can avoid losing money if they press the button quickly enough. Finally, on ‘neutral’ trials, the participant responded in a similar way, but no money was involved. For more details, please see papers on the overview of the ABCD study (Casey et al., 2018, Chaarani et al., 2021, Hagler Jr et al., 2019). The ventral striatum has been highlighted as a key neural correlate of reward processing in the MID task (Beck et al., 2009, Cao et al., 2019, Casey et al., 2018, Knutson et al., 2001, Knutson and Heinz, 2015). Therefore, the current study employed a region-of-interest (ROI) approach by examining ventral striatum activity during reward anticipation and reward receipt. The left and right hemispheres of the brain were averaged in assessing the ventral striatum ROI. Freesurfer’s anatomically-defined parcellations were mapped onto each individual’s cortical surface space to derive ventral striatum activity (Fischl et al., 2002). Activity during reward anticipation was measured by the contrast between the anticipation of a reward and the anticipation of a neutral outcome. Activity during reward receipt was measured by the contrast between positive reward feedback and negative reward feedback. Estimates of ventral striatum activity related to each of these contrasts were used in the subsequent analyses.

### 2.3 fMRI data acquisition and preprocessing

The ABCD study used a harmonized neuroimaging protocol across 21 sites. Three 3 T scanner platforms (i.e., Siemens Prisma [Siemens Healthineers], GE 750 [GE Healthcare], and Philips [Philips Healthcare]) were used. For Siemens scanners, the following scanning parameters were used for T1 structural image acquisition: matrix = 256 × 256, 176 slices, field of view (FOV) = 256 × 256, resolution (mm) = 1.0 × 1.0 × 1.0, TR = 2500 ms, TE = 2.88 ms, TI = 1060 ms, flip angle = 8°. For Phillips scanners, the following scanning parameters were used for T1 structural image acquisition: matrix = 256 × 256, 225 slices, field of view (FOV) = 256 × 240, resolution (mm) = 1.0 × 1.0 × 1.0, repetition time (TR) = 6.31 ms, echo time (TE) = 2.9 ms, inversion time (TI) = 1060 ms, flip angle = 8°. For GE scanners, the following scanning parameters were used for T1 structural image acquisition: matrix = 256 × 256, 208 slices, field of view (FOV) = 256 × 256, resolution (mm) = 1.0 × 1.0 × 1.0, TR = 2500 ms, TE = 2 ms, TI = 1060 ms, flip angle = 8°. Across all scanners, the following scanning parameters were used for T2 * weighted functional images associated with the MID task: matrix = 90 × 90, 60 slices, FOV = 216 × 216, TE/TR (ms) = 30/800, flip angle = 52°, resolution (mm) = 2.4 × 2.4 × 2.4, multiband acceleration factor = 6. Each scanner used a standard head coil for the initial time point of fMRI data acquisition.

The MID task was presented to participants in a random order along with other functional tasks included in the study. Automated and manual methods were used to assess the quality of raw fMRI images, which looked for problems with acquisition, artifacts, motion, or file corruption. Subsequent preprocessing of these images removed initial frames of functional images. The pipeline estimated within-volume head motion and performed rigid body motion correction in each individual. Data were processed for image distortions resulting from B0 field inhomogeneity. Isotropic resampling (2.4 mm) aligned fMRI data across participants from all sites. Functional data were registered to each individual’s T1-weighted structural image. Following preprocessing, images are sampled onto the cortical surface of each individual subject using FreeSurfer functions (Hagler et al., 2019). General linear modeling using AFNI’s 3dDeconvolve (Cox, 1996) was used to calculate individual-level models. Baseline and quadratic trends in time-series data were included in all first-level analyses. Motion estimates and their derivatives were also included in individual level models as regressors of no interest (Power et al., 2014). In cases where a single time point was associated with FD greater than 0.9, this volume was censored. Estimates were filtered with an infinite impulse response notch filter, which attenuates signals in the range of 0.31–0.43 Hz. This filtering is thought to result in motion estimates and FD values that more accurately reflect head motion (Fair et al., 2020). A two-parameter gamma basis function was convolved with onsets of each MID task event during the anticipation and outcome phases of the task.

### 2.4 Questionnaire measures

#### 2.4.1 Parents’ familism values

At T1, parents’ familism values were measured using three familism-related subscales of the Mexican American Cultural Values Scale (MACVS; Knight et al., 2010). On a five-point Likert scale (from 1 = *not at all* to 5 = *completely*), parents reported on the extent they agree with beliefs on family support, family obligation, and family as referent. Family support subscale includes six items reflecting emotional reliance on and intimacy with family (e.g., “It is important for family members to show their love and affection to one another”; *α* = .80). Family obligations subscale includes five items reflecting the responsibilities to provide help to family members when needed (e.g., “If a relative is having a hard time financially, one should help them out if possible”; *α* = .71). Family as referent subscale includes five items reflecting the preference to consider family as an important reference group when making decisions (e.g., “A person should always think about their family when making important decisions”; *α* = .74). Following prior research (e.g., Armenta et al., 2011; Knight et al., 2016; Stein et al., 2020), mean scores were taken across items in each subscale, and a latent construct of familism values was generated with the three subscale scores as indicators.

#### 2.4.2 Youth’s prosocial behaviors

At T1 and T2, youth’s prosocial behaviors were assessed using three items adapted from the Prosocial Behaviors subscale of the Strengths and Difficulties Questionnaire (Goodman et al., 1998). On a three-point Likert scale (from 0 = *not true* to 2 = *certainly true*), parents rated how true each item described their children (e.g., “My child is considerate of other people’s feelings”, “My child is helpful if someone is hurt, upset, or feeling ill”; *α* = .78 at T1 and .80 at T2). The mean score of the items was calculated to indicate youth’s prosocial behaviors, with higher scores indicating higher levels of prosocial behaviors.

#### 2.4.3 Demographic covariates

In line with prior research using the ABCD data (e.g., Barch et al., 2021; Karcher et al., 2021; Lees et al., 2021), the current study included youth’s age, biological sex, parents’ educational attainment, and household financial adversity as demographic covariates. Youth’s biological sex was coded into 0 = *male* and 1 = *female*. Parents’ educational attainment was the highest educational degree in the family, ranging from 1 = *less than a high school diploma* to 5 = *postgraduate degree*. Household financial adversity was assessed using the Parent-Reported Financial Adversity Questionnaire (PRFQ) (Diemer et al., 2013), which was the sum score on experiences of financial difficulties in the past 12 months (7 items, 0 = *no* and 1 = *yes*, range = 0–7; e.g., “In the past 12 months, has there been a time when you and your immediate family didn’t pay the full amount of the rent or mortgage because you could not afford it?”). Given that the current study focused on Latinx American parents’ familism values, parents’ gender and nativity were also included. Parents’ gender was coded into 0 = *male* and 1 = *female*. Parents’ nativity was coded into 0 = *born in the United States*, 1 = *born outside of the United States*.

#### 2.4.4 Overview of the analyses

Descriptive statistics and bivariate correlations were first conducted. The primary analyses included three sets of Structural Equation Modeling (SEM) models to test the hypotheses using Mplus 8.9. The attrition rate from T1 to T2 was 14%. The Little’s MCAR test suggested that the data were not missing completely at random (chi-square = 117.59, *p* < .001; Little, 1988). Therefore, maximum likelihood estimation with robust standard errors (MLR), which is an estimator robust to non-normality and non-independence (Kline, 2015), was used to handle missing data and provide unbiased standard errors. To account for the nested structure of the sampling with siblings within a family, the Taylor series linearization using the TYPE = COMPLEX command in Mplus was applied to all SEM models. As for the clustering effect derived from the multisite design, the STRATIFICATION = SITE ID command in Mplus was used to adjust for the estimated parameters by taking into account the non-independence of the observations. Three goodness-of-fit statistics were reported and used to evaluate the model fit: (a) the comparative fit index (CFI) > .90, (b) the root-mean-square error of approximation (RMSEA) < .08, and (c) the standardized root-mean-square residual (SRMR) < .06 (Hu and Bentler, 1999).

Following the pre-registered analytic plan (https://aspredicted.org/YCL_PBR), the first set of analyses examined the main effect of parents’ familism values on youth’s prosocial behaviors over time using a SEM model. Parents’ familism values were specified as a latent variable with three indicators, that is, family support, family obligation, and family as referent. Youth’s prosocial behaviors at T2 were predicted by parents’ familism values at T1, controlling for youth’s prosocial behaviors at T1 and demographic covariates.

The second set of analyses tested the moderating role of youth’s neural reward sensitivity on the longitudinal association between parents’ familism values and youth’s prosocial behaviors. Youth’s prosocial behaviors at T2 were predicted by parents’ familism values at T1, youth’s neural reward sensitivity at T1, and parents’ familism values × youth’s neural reward sensitivity at T1, controlling for youth’s prosocial behaviors at T1 and other covariates. An SEM model was run for each of the two neural reward sensitivity variables (i.e., ventral striatum activity during reward anticipation/receipt). To generate the interaction term involving both an observed variable (i.e., youth’s neural sensitivity) and a latent variable (i.e., parents’ familism values), the latent moderated structural equations (LMS) approach was adopted using the XWITH command in Mplus (Maslowsky et al., 2015, Ip et al., 2022). The typical goodness-of-fit indexes are not available in LMS models. Therefore, the fit indexes before adding the latent interaction term were used to demonstrate the adequate model fit of LMS models following recommended practices (Poteat et al., 2021, Zhou et al., 2022). For all significant interactions, the effects were probed using the simple slope technique (Bauer and Curran, 2005), which presents the associations between parents’ familism values and youth’s prosocial behaviors among youth with low (i.e., 1 SD below the mean) and high (1 SD above the mean) ventral striatum activity during reward processing. In Mplus, the simple slopes were estimated by applying model constraints of fixing the moderator to 1 SD above and below the mean. Moreover, Roisman indices were estimated to examine whether the interaction effects align with the theory of differential susceptibility to environmental influences (Roisman et al., 2012). Following the practice of prior research (e.g., Deane et al., 2020), the regions of significance (RoS) on X, the proportion of the interaction (PoI), and the proportion affected (PA) were calculated.

The third set of analyses tested the moderating role that youth’s baseline prosocial behaviors may play in the link between parents’ familism values and youth’s prosocial behaviors one year later. Youth’s prosocial behaviors at T2 were predicted by parents’ familism values at T1, youth’s prosocial behaviors at T1, and parents’ familism values × youth’s prosocial behaviors at T1, controlling for demographic covariates. Similar procedures and principles were followed to generate an LMS model involving the interaction term between a latent variable (i.e., parents’ familism values) and an observed variable (i.e., youth’s baseline prosocial behavior). Again, simple slope analyses were used to probe the conditional associations between parents’ familism values and youth’s prosocial behaviors among youth with low (i.e., 1 SD below the mean) and high (1 SD above the mean) baseline prosocial behaviors.

Finally, supplementary analyses were conducted to ensure that the findings were specific to the ventral striatum. Given that other brain regions (e.g., dorsal striatal and prefrontal regions) also play important roles in reward processing (for a review, see O’doherty, 2004), these supplementary analyses investigated whether the activities in the dorsal striatum and the orbitofrontal cortex (OFC) during reward processing moderate the associations between parents’ familism values and youth’s prosocial behaviors over time. Specifically, youth’s neural activities in caudate, putamen, lateral OFC, and medial OFC during both reward anticipation and reward receipt were included. The pre-registration only included the ventral striatum as a region of interest, and these supplementary analyses on dorsal striatum and OFC activity were exploratory.

## 3 Results

### 3.1 Descriptive statistics and bivariate correlations

Table 1 shows the descriptive statistics and correlations between key variables included in the present study. As components of parents’ familism values, family support, family obligation, and family as referent were highly correlated with each other (*r*s > .62, *p*s < .001). Parents’ familism values were positively correlated with youth’s prosocial behaviors at both T1 and T2 (*r*s > .08, *p*s < .01). Youth’s ventral striatum activity during reward anticipation was not correlated with such activity during reward receipt, and both were generally not correlated with their prosocial behaviors except a small correlation between T1 ventral striatum during reward receipt and T2 prosocial behaviors (*r* = .05, *p* = .05). Correlations between key variables and demographic covariates were also examined. Girls had higher levels of prosocial behaviors at both T1 and T2 (*r*s > .12, *p*s < .001). Additionally, youth’s age was negatively correlated with prosocial behaviors at T2 but not at T1 (*r* = −.08, *p*s = .002). Parents’ gender, nativity, educational attainment, and families’ financial adversity were not correlated with youth’s prosocial behaviors at T1 or T2.

**Table 1:** Descriptive Statistics and Correlations Among Key Variables.

	1	2	3	4	5	6	7
1. T1 parents’ familism values of family support	--						
2. T1 parents’ familism values of family obligation	.65^***^	--					
3. T1 parents’ familism values of family as referent	.62^***^	.73^***^	--				
4. T1 youth’s VS during reward anticipation	-.06*	-.02	-.02	--			
5. T1 youth’s VS during reward receipt	-.01	-.03	.00	.02	--		
6. T1 youth’s prosocial behaviors	.11^***^	.08^**^	.09^***^	.01	.03	--	
7. T2 youth’s prosocial behaviors	.11^***^	.09^***^	.09^***^	-.00	.05*	.45^***^	--
*Mean*	4.37	3.83	3.63	.05	.14	1.79	1.74
*SD*	.57	.69	.79	.26	.31	.37	.40
*Min*	1.83	1.60	1.00	-2.49	-2.68	.00	.00
*Max*	5.00	5.00	5.00	1.67	2.64	2.00	2.00
*Skewness*	-.93	-.14	-.36	-.20	-.25	-2.00	-1.64
*Kurtosis*	.68	-.55	-.21	9.65	8.89	4.16	2.15

*Note.* VS = ventral striatum.

* *p* < .05. ^**^p < .01. ^***^p < .001.

### 3.2 Main effect of parents’ familism values on youth’s prosocial behavior

The first set of analyses was to examine the main effect of parents’ familism values on youth’s prosocial behaviors over time. The model fit of the main effect model was adequate, CFI = .97, RMSEA = .05, SRMR = .02. The three indicators (i.e., family support, family obligations, family as referent) loaded significantly on the latent variable of parents’ familism values with the factor loadings ranging from.75 to.86. The results showed that parents’ familism values were associated with youth’s higher levels of prosocial behaviors one year later, controlling for their baseline prosocial behaviors and the demographic covariates, β = .08, *p* = .005.

### 3.3 The moderating role of youth’s neural activity during reward processing

**Fig. 1:**
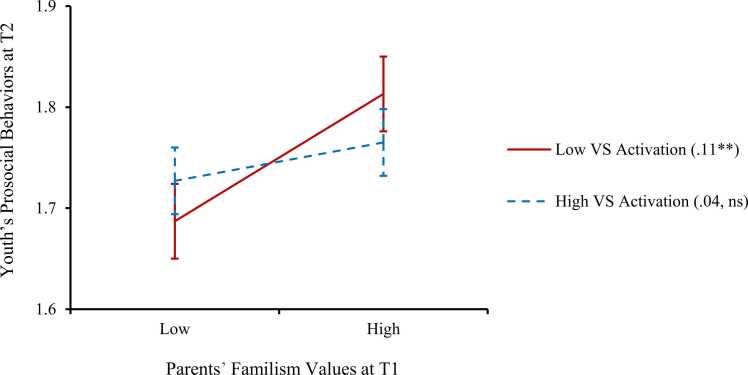
The association between parents’ familism values at T1 and youth’s prosocial behaviors at T2 was moderated by youth’s ventral striatum (VS) activation during reward anticipation. *Note.* Low (or high) VS activation and familism values are 1 SD below (or above) the mean. Error bars indicate the 95% confidence interval of the estimation. Unstandardized simple slopes were presented in the parentheses. ** *p* < .01. ns = not significant.

The second set of analyses was to test the moderating role of youth’s ventral striatum activity during reward processing on the longitudinal association between parents’ familism values and youth’s prosocial behaviors. The two moderation models (i.e., one for reward anticipation and the other for reward receipt) showed good model fits, CFIs > .97, RMSEAs < .05, SRMRs < .02, which were estimated using the fit indices before entering the latent interaction term. The interaction effect between youth’s ventral striatum activity during reward anticipation and parents’ familism values on youth’s prosocial behaviors over time was significant (β = −.04, *p* = .04; Model 1 of Table 2). Simple slope analyses were used to disentangle the associations between parents’ familism values at T1 and youth’s prosocial behaviors at T2 for youth with low (i.e., M–1 SD) versus high (i.e., M+1 SD) ventral striatum activity during reward anticipation (Fig. 1). For youth who showed low neural activity, parents’ familism values were associated with youth’s higher levels of prosocial behaviors one year later (unstandardized simple slope =.11, *p* = .001). For youth who showed high neural activity, there was no significant relation between parents’ familism values and youth’s later prosocial behaviors (unstandardized simple slope =.04, *p* = .24). The moderation model of youth’s ventral striatum activity during reward receipt was also examined. However, there was no significant moderating effect of ventral striatum activity during reward anticipation (β = .00, *p* = .90; Model 2 of Table 2) on the link between parents’ familism values and youth’s later prosocial behaviors.

**Table 2:** Moderation Effects of Youth’s Neural Reward Sensitivity on the Link Between Parents’ Familism Values and Youth’s Prosocial Behaviors.

	Predicting youth’s prosocial behavior at T2						
	Model 1: VS reward anticipation				Model 2: VS reward receipt		
	*b*	*SE*	β		*b*	*SE*	β
Youth’s prosocial behaviors at T1	.49	.04	.45^***^		.49	.04	.45^***^
Parents’ familism values at T1	.07	.03	.08^**^		.07	.03	.07^**^
Youth’s VS activation at T1	-.01	.04	.01		.04	.03	.03
Parents’ familism values × VS activation at T1	-.15	.07	-.04*		.01	.07	.00
Covariates							
Youth’s age	-.04	.01	-.06^**^		-.04	.02	-.06^**^
Youth’s biological sex	.03	.02	.04		.03	.02	.04
Parents’ gender	-.10	.03	-.07^**^		-.09	.03	-.07^**^
Parents’ nativity	.02	.02	.03		.02	.02	.03
Parents’ education	.02	.01	.06*		.02	.01	.06*
Household financial adversity	-.01	.01	-.02		-.01	.01	-.02

*Note. b* = unstandardized coefficient, *SE* = standard error of *b*, β = standardized coefficient. VS = ventral striatum. For youth biological sex and parent gender, 0 = *male*, 1 = *female*; for parent nativity, 0 = *born in the US*, 1 = *born outside of the US*; parent educational attainment ranges from 1 = *less than a high school diploma* to 5 = *postgraduate degree*.

* p < .05. * * p < .01. * ** p < .001.

Roisman indices (i.e., RoS on X, PoI, and PA) were calculated to examine whether the interactive role of parents’ familism values and youth’s ventral striatum activity during reward anticipation in youth’s prosocial behaviors aligns with the theory of differential susceptibility to environmental influences. The RoS on X had a lower bound of − 1.50 and an upper bound of 1.62. Given that the RoS was within the + /–2 SD of the mean of X (i.e., parents’ familism values), the interaction effect aligned with the differential susceptibility model, such that youth with low vs. high ventral striatum activity during reward anticipation were significantly different in prosocial behaviors at both low and high levels of parents’ familism values. PoI of the interaction effect was 56%, which was highly consistent with differential susceptibility (i.e., within the range of 40% to 60%). The PA of the interaction effect was 54%, which was also highly consistent with differential susceptibility (i.e., close to the prototypical value of 50%).

### 3.4 The moderating role of youth’s baseline prosocial behaviors

**Fig. 2:**
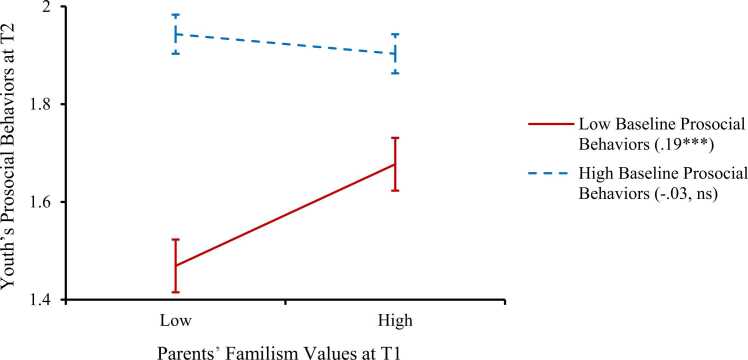
The association between parents’ familism values at T1 and youth’s prosocial behaviors at T2 was moderated by youth’s prosocial behaviors at T1. *Note.* Low (or high) baseline prosocial behaviors and familism values are 1 SD below (or above) the mean. Error bars indicate the 95% confidence interval of the estimation. Unstandardized simple slopes were presented in the parentheses. *** *p* < .001. ns = not significant.

The third set of analyses was to test the moderating role of youth’s baseline prosocial behaviors in the longitudinal link between parents’ familism values and youth’s prosocial behaviors. The moderation model of youth’s baseline prosocial behaviors before entering the interaction term showed good model fit, CFI = .97, RMSEA = .05, SRMR = .02. As shown in Table 3, the results revealed a significant interaction effect between youth’s baseline prosocial behaviors and parents’ familism values on youth’s prosocial behaviors one year later, β = −.12, *p* = .005. As shown in Fig. 2, simple slope analyses suggested that for youth who showed lower levels of baseline prosocial behaviors, parents’ familism values significantly predicted higher levels of prosocial behaviors one year later (unstandardized simple slope = .19, *p* < .001). In contrast, for youth who showed higher levels of baseline prosocial behaviors, parents’ familism values were not associated with youth’s later prosocial behaviors over time (unstandardized simple slope = −.03, *p* = .35).

**Table 3:** Moderation Effects of Baseline Youth’s Prosocial Behaviors on the Link Between Parents’ Familism Values and Youth’s Prosocial Behaviors.

	Predicting youth’s prosocial behavior at T2		
	*b*	*SE*	β
Youth’s prosocial behaviors at T1	.48	.04	.44^***^
Parents’ familism values at T1	.08	.03	.08^**^
Parents’ familism values × Youth’s prosocial behaviors at T1	-.30	.11	-.12^**^
Covariates			
Youth’s age	-.04	.01	-.06^**^
Youth’s biological sex	.03	.02	.04
Parents’ gender	-.09	.03	-.06^**^
Parents’ nativity	.03	.02	.03
Parents’ education	.02	.01	.06*
Household financial adversity	-.01	.01	-.02

*Note. b* = unstandardized coefficient, *SE* = standard error of *b*, β = standardized coefficient. For youth biological sex and parent gender, 0 = *male*, 1 = *female*; for parent nativity, 0 = *born in the US*, 1 = *born outside of the US*; parent educational attainment ranges from 1 = *less than a high school diploma* to 5 = *postgraduate degree*.

* *p* < .05. ^**^p < .01. ^***^p < .001.

Given that both youth’s ventral striatum activity during reward anticipation and youth’s baseline prosocial behaviors moderated the longitudinal association between parents’ familism values and youth’s prosocial behaviors, an additional model that included both moderators was conducted to examine whether these moderators interfere with each other. Results suggested that, after including both interaction terms simultaneously in the same model, the interaction between parents’ familism values and youth’s ventral striatum activity during reward anticipation (β = −.04, *p* = .04) and the interaction between parents’ familism values and youth’s baseline prosocial behaviors (β = −.12, *p* = .007) still remain significant.

### 3.5 Supplementary analyses

Additional analyses were conducted to examine if the moderation effects were specific to ventral striatum activity during reward processing. The same set of moderation models were conducted with the dorsal striatum and the OFC activity during reward anticipation and receipt. The results indicated that youth’s caudate (β = −.00, *p* = .86), putamen (β = −.02, *p* = .32), lateral OFC (β = −.02, *p* = .39), and medial OFC (β = −.02, *p* = .53) activity during reward anticipation did not moderate the longitudinal association between parents’ familism values and youth’s prosocial behaviors. Similarly, youth’s caudate (β = −.02, *p* = .44), putamen (β = −.02, *p* = .44), lateral OFC (β = .00, *p* = .99), and medial OFC (β = .02, *p* = .39) activity during reward receipt also did not moderate the longitudinal association between parents’ familism values and youth’s prosocial behaviors.

## 4 Discussion

In the current research, parents’ familism values were associated with increased prosocial behaviors among Latinx American youth over two years during early adolescence. This finding is consistent with prior concurrent and longitudinal studies probing the link between parents’ familism values and Mexican American youth’s prosocial tendencies (Calderón-Tena et al., 2011, Knight et al., 2016). With nationally representative data from the ABCD study, the current study confirmed the role of parents’ familism values in youth’s prosocial development. Parents’ familism values may influence youth’s prosocial behaviors in several ways. First, the more parents endorse familism values, the more they are willing to socialize such values to youth (Knight et al., 2011). Such cultural socialization is key in shaping Latinx American youth’s internalization of familism values (Knight et al., 1993, Umaña‐Taylor et al., 2009), which is consistently related to their greater prosocial tendencies (Knight et al., 2015, Knight et al., 2018). Second, parents who endorse greater familism values are more likely to directly ask for youth’s help with regard to family chores and caregiving, and such specific parenting practice on prosocial expectations may in turn contribute to youth’s greater prosociality (Calderón-Tena et al., 2011). Finally, parents with greater familism beliefs are more likely to get involved in youth’s development and make personal sacrifices to help youth (Cheah et al., 2012, Padilla et al., 2020, Stein et al., 2014). When parents demonstrate prosocial behaviors through their support to youth, it may promote youth’s own prosocial behaviors over time. Neural reward sensitivity on the other hand was not associated with prosocial behaviors. Although there was a small correlation between ventral striatum activity during reward receipt and later prosocial behaviors, the association was no longer significant after adjusting for baseline prosocial behaviors. This is consistent with past research which suggests that only neural activation to monetary reward for others, but not such activation to monetary for oneself, promotes prosocial behaviors (Morelli et al., 2018). Past research on self-reported reward responsiveness also indicates that reward responsiveness does not contribute to adolescents’ prosocial development (Blankenstein et al., 2020).

Echoing the call of incorporating culture into the study of brain development (Qu et al., 2021), the current study is, to the best of our knowledge, the first to examine the interactive role of cultural value and brain development in youth’s prosocial development. In line with our hypotheses, youth’s ventral striatum activity during reward anticipation moderated the link between parents’ familism values and Latinx American youth’s prosocial behaviors, such that parents’ familism values were only associated with increased prosocial behaviors over time when youth showed a lower level of neural reward sensitivity. The Roisman indices (Roisman et al., 2012) showed that the interactive role of parents’ familism values and youth’s neural reward sensitivity in Latinx American youth’s prosocial behaviors is in line with the theory of differential susceptibility to environmental influences (Belsky et al., 2007, Ellis and Boyce, 2011, Guyer, 2020, Schriber and Guyer, 2016). Heightened neural reward sensitivity may reflect youth’s worse relationships with parents (Casement et al., 2014, Qu et al., 2015), such that they are more attuned to monetary reward outside the family to compensate for the lack of social rewards at home. In this case, Latinx American youth with heightened neural reward sensitivity may be less receptive to parents’ cultural socialization, such that their prosocial behaviors are less likely to be influenced by parents’ endorsement of cultural values.

Another explanation of this interaction is the poor fit between brain development and cultural environment. Given that familism places a strong emphasis on providing support and fulfilling obligation to other members of the family (Sabogal et al., 1987), greater neural sensitivity during the anticipation of monetary gain for oneself may suggest that youth are less receptive to such cultural values. For youth who show greater sensitivity to reward, because familism has a focus on *others* and reward sensitivity has a focus on *self*, there may be a poor fit between their brain development and cultural environment, which hinders the positive influence of parents’ familism values on their prosocial development. In contrast, lower neural sensitivity during reward anticipation amplifies the impact of parents’ familism values on Latinx American youth’s prosocial behavior. Youth who are less attuned to reward may be more receptive to parents’ familism values and related socialization of values, and thus they may be more likely to be influenced by their parents’ familism values. In this case, lower neural reward sensitivity marks Latinx American youth’s high susceptibility to parents’ familism values, such that youth with lower reward sensitivity would show developmental enhancement in an environment of high familism values and developmental vulnerability in an environment of low familism values.

However, youth’s ventral striatum activity during reward *receipt* did not moderate the link between parents’ familism values and youth’s prosocial behaviors. Such difference in the moderating roles of reward anticipation and reward receipt may happen because, despite both being essential to reward processing, their neural processes are distinct from each other. The differences were also reflected in their correlations in the current study, given that ventral striatum activity during reward anticipation was not correlated with such activity during reward receipt. Whereas anticipation emphasizes the processing of initial encounter of the prospect of reward, receipt emphasizes the processing of reward-related results (Oldham et al., 2018). Past studies suggest that there are significant differences in the neural response between anticipation and receipt of reward (Pornpattananangkul and Nusslock, 2015, Simon et al., 2015), and their developmental trajectories also differ during adolescence (Hoogendam et al., 2013). In the current study, youth’s ventral striatum activity during reward anticipation and reward receipt also showed different correlations with parents’ familism values. Whereas ventral striatum during reward anticipation was negatively associated with parents’ endorsement of family support, such activity during reward receipt was not associated with any component of familism values. A possible explanation for this difference is that reward anticipation in the MID task is a complicated phase that involves uncertainty (i.e., whether the participant is able to win the reward), which is an important component of risk attitude (Peterman and Anderson, 1999). Past studies suggest that youth who endorse familism values take fewer risks (Wheeler et al., 2017). Therefore, it is possible that heightened neural response to reward anticipation (vs. reward receipt) resembles a larger contrast with familism values. Given such a larger contrast, youth with heightened neural response to reward anticipation may be less receptive to parental socialization of familism values, and thus their prosocial behaviors are less likely to be influenced by parents’ familism values. Nevertheless, the findings are not consistent and comprehensive enough to conclude the difference in how reward anticipation and reward receipt relate to cultural values and prosocial development. Future studies can include more measures of cultural values to examine how reward anticipation and reward receipt contribute to cultural transmission.

As expected, youth’s baseline prosocial behaviors also moderated the longitudinal link between parents’ familism values and youth’s prosocial behaviors two years later. Parents’ familism values predicted youth’s increased prosocial behaviors over time only when youth reported a lower baseline level of prosocial behaviors, but not when youth reported a higher baseline level of prosocial behaviors. The results are consistent with previous literature showing that youth who show a lower level of prosocial behaviors initially may benefit more from positive parents’ beliefs and parenting practices that can provide supportive resources (Pomerantz et al., 2007, Zhou et al., 2022). In contrast, youth who have shown a high level of prosocial behaviors at baseline may already possess adequate resources that are important for such positive development, and therefore, are influenced less by additional parental support in this process. In addition, the youth sample in the current study had an average score over 1.7 on a scale ranging from 0 to 2 on the prosocial behaviors measure. This indicates that there may be a ceiling effect that limits youth who initially had a high level of prosocial behaviors to show significant increment over time, even though they may also have internalized parent’s familism values.

## 5 Limitation and future directions

The current study has several limitations. First, although the findings of the current study were derived from longitudinal design, they were based on correlational data and thus did not allow for causal conclusions. Second, both familism values and prosocial behaviors were reported by parents, and thus the results may be influenced by same-rater bias. Third, the current study only examined youth’s neural activation during general reward processing in the MID task, and thus it is unclear how their neural response to social reward (e.g., parental approval) and prosocial reward (e.g., reward for others) may play a role in the link between cultural environment and prosocial development. Finally, the current study only examined youth’s prosocial development during early adolescence, which left an open question of whether similar findings apply to youth during mid- and late adolescence.

These limitations point to directions for future research. First, it is important for future studies to incorporate youth’s own endorsement of familism values to examine how Latinx American youth differ in their susceptibility to parents’ familism values. Similarly, when examining youth’s prosocial behaviors, future studies should assess multiple raters’ reports (e.g., youth’s report and teachers’ report) to avoid same-rater bias. Second, it is crucial for future research on youth’s susceptibility to cultural environments to expand beyond the examination of general neural reward sensitivity. For example, future studies can examine a diverse range of neural reward sensitivity using tasks on social vs. non-social reward (Lin et al., 2012), family reward vs. personal reward (Telzer et al., 2010), and prosocial reward vs. personal reward (Telzer et al., 2014). Neural sensitivity to social, family, or prosocial reward may reflect coherence with familism values. Therefore, it is possible that youth with high neural sensitivity to social, family, and prosocial reward would be more likely to be influenced by parents’ cultural socialization. Finally, given that youth may show a declined trajectory in prosocial behaviors across adolescence (Luengo Kanacri et al., 2013), it is important for future studies to examine the role of cultural values in Latinx American youth’s prosocial development during different phases of adolescence and investigate how such roles change over the course of adolescence.

## 6 Conclusions

Research on Latinx American youth suggests that parents’ familism values play a positive role in their prosocial development. However, little is known about what neural and behavioral factors may contribute to individual variability in this developmental process. Using a large-scale longitudinal sample, our results suggest that parents’ familism values play a larger role in promoting Latinx American youth’s prosocial behaviors when the youth are less attuned to reward at the neural level. Moreover, for Latinx American youth who show lower levels of prosocial behaviors initially, their parents’ familism values have a stronger association with their prosocial behaviors two years later. Taken together, the findings of the current study highlight individual differences in neurobiological and behavioral development as markers of sensitivity to cultural environments with regard to Latinx American youth’s prosocial development. By demonstrating the role of familism in prosocial development and youth’s susceptibility to such cultural environment, our findings point to the importance of developing culturally informed interventions for Latinx American youth and also help lay the foundation for identifying groups of youth who may benefit more from such interventions. Ultimately, the findings imply the necessity of developing strengths-based policies and interventions which support Latinx American youth’s prosocial development.

## CRediT authorship contribution statement

**Beiming Yang:** Writing – review & editing, Writing – original draft, Visualization, Software, Methodology, Formal analysis, Data curation, Conceptualization. **Zexi Zhou:** Writing – review & editing, Writing – original draft, Software, Methodology, Formal analysis, Conceptualization. **Varun Devakonda:** Writing – review & editing. **Yang Qu:** Writing – review & editing, Supervision, Resources, Methodology, Funding acquisition, Conceptualization.

## Declaration of Competing Interest

The authors have no conflict of interest to declare.

## Data Availability

Data were obtained from baseline and two-year follow-up of the Adolescent Brain Cognitive Development (ABCD) study (data release 4.0). All the data included in the current study are available on the NIMH Data Archive (https://nda.nih.gov/abcd) upon data access request.
